# Sjögren’s Syndrome Masquerading as Testicular Vasculitis

**DOI:** 10.31138/mjr.250323.ssm

**Published:** 2024-05-21

**Authors:** Rajaie Namas, Hamdan Alawadhi, Priti Joshi, Esat Memisoglu

**Affiliations:** 1 Department of Rheumatology, Medical Subspecialities Institute, Cleveland Clinic Abu Dhabi; 2 Department of Pathology, Pathology & Laboratory Medicine Institute, Cleveland Clinic Abu Dhabi; 3 Department of Radiology, Imaging Institute, Cleveland Clinic Abu Dhabi

**Keywords:** Sjögren’s syndrome, vasculitis

A 49-year-old man presented to the outpatient rheumatology clinic to be evaluated for an underlying connective tissue disease in the context of polyarthritis and right painful testicular mass. The patient had a two-month history of right testicular pain and felt a mass involving his right testicle. He also endorsed a history of polyarthritis, fatigue, decreased salivary pooling, and ocular dryness.

Laboratory findings revealed a white cell count was 11.7 x109/L (normal 4.5 – 11 x109/), haemoglobin 132 g/l (normal 131–172 g/L), platelets 276 x109/L (Normal 140–400 x109/), C-reactive protein 1 mg/L (normal 5 mg/L), and erythrocyte sedimentation rate 55 mm/hr (normal 15 mm/hr). He had an elevated serum anti-SSA antibody of 2.5 U (normal range 1 U) with normal levels of SS-B/La antibodies of 0.2U (normal 1) and Anti-Smith antibodies, raising the possibility for Sjögren’s syndrome. Otherwise, his rheumatological workup was unremarkable, including rheumatoid factor <10 (normal ≤13 IU/ml), anti-cyclic citrullinated peptide antibody <7 u/mL (normal ≤16U/mL), immunofluorescence antinuclear antibody <1:80 (Normal <1:80), anti-myeloperoxidase antibody <0.2 (normal <0.4), anti-cytoplasmic neutrophilic antibody (ANCA), anti-proteinase 3 antineutrophil cytoplasmic antibody (PR3-ANCA) (normal <0.4), antiphospholipid profile and anti-double-stranded DNA <12.3 IU/mL (normal range<30). Normal complement levels, protein electrophoresis and angiotensin converting enzyme levels 37 U/L (normal 14 - 82 U/L). Infectious workup, including Brucella testing, has also been inconclusive. He was also evaluated by ophthalmology and was found to have a positive Schirmer test. A computed tomography scan of the chest, abdomen, and pelvis revealed a mass above the right testis at the right distal spermatic cord **(Figure 1)**. His ultrasound of the parotids, on the other hand, was unremarkable. The patient had a radical orchiectomy, and the surgical pathology revealed granulomatous and giant cell inflammation in the spermatic cord, forming a mass-like lesion, as well as vasculitis and no evidence of neoplasia **(Figure 2)**. Mycobacterium tuberculosis PCR from testicular tissue, as well as fungal and bacterial cultures, all came back negative.

**Figure 1.**
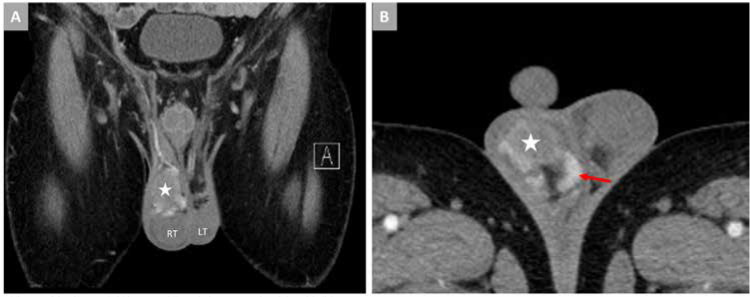
**(A)** Coronal CT scan of the abdomen and pelvis with IV contrast: Mass (3.7 x3x2.4 cm) in the distal right spermatic cord seen above the right testis. Asterisk: mass; RT: right testis; LT: left testis. **(B)** Axial CT section at the level of mass lesion. Red arrow: contrast enhancing varicoceles.

**Figure 2.**
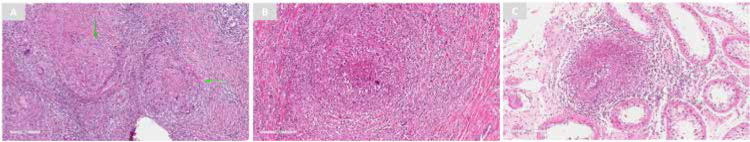
**(A)** High-power (40X) view of spermatic cord section showing granulomatous inflammation (arrows) and giant cells. **(B)** A High-power (40X) view of testis section away from site of inflammation showing a focus small vessel arteritis (fibrinoid damage of vessel wall with lymphocytic infiltrate). **(C)** A High-power (40X) view of epididymis section showing a focus of small vessel arteritis (fibrinoid necrosis and lymphocytic infiltrate in vessel wall).

Based on the clinical presentation, as well as the positive anti-SSA antibody and pathology results, he was diagnosed with Sjögren’s syndrome associated testicular vasculitis. As a result, the patient was commenced on oral hydroxychloroquine and methotrexate, which resulted in significant improvement of his joint symptoms as well as no new onset testicular symptoms on subsequent follow-up visits. Because of its rarity, testicular vasculitis has received little attention in the scientific literature.^1,2^ To the best of our knowledge, this is the first case of Sjögren’s syndrome-related testicular vasculitis reported in English literature. This clinical case emphasises the atypical clinical presentation of Sjögren’s syndrome, raising clinicians’ awareness of the importance of maintaining a high clinical suspicion when dealing with patients with vasculitis due to its variable clinical presentation.

## References

[1] Dixit A Hague C Bicknell S. Testicular Vasculitis: A Sonographic and Pathologic Diagnosis. Case Rep Radiol 2017;2017:8923621. doi:10.1155/2017/8923621 28246567 PMC5299182

[2] Lintern N Johnson NR Mckenzie I Martin B. Testicular vasculitis - literature review and case report in Queensland. Curr Urol 2013;7(2):107–9. doi:10.1159/000356258 24917768 PMC4017738

